# Hybrid optimization for efficient 6G IoT traffic management and multi-routing strategy

**DOI:** 10.1038/s41598-024-81709-z

**Published:** 2024-12-28

**Authors:** J. Logeshwaran, Shobhit K. Patel, Om Prakash Kumar, Fahah Ahmed Al-Zahrani

**Affiliations:** 1 Department of Computer Science, Christ University, Bengaluru, Karnataka 560029 India; 2 Department of Computer Engineering, Marwadi University, Rajkot, 360003 India; 3 Department of Electronics and Communication Engineering, Manipal Institute of Technology, Manipal Academy of Higher Education, Manipal, 576104 India; 4 Computer Engineering Department, Umm Al-Qura University, Mecca, 24381 Saudi Arabia

**Keywords:** Air pollution monitoring, IoT, Mobile ad-hoc networking, Quantum-inspired clustering algorithm, Quantum entanglement and mobility metric, Deep reinforcement learning, Dynamic cluster head selection, Quantum genetic algorithm, Computer science, Information technology, Electrical and electronic engineering

## Abstract

Efficient traffic management solutions in 6G communication systems face challenges as the scale of the Internet of Things (IoT) grows. This paper aims to yield an all-inclusive framework ensuring reliable air pollution monitoring throughout smart cities, capitalizing on leading-edge techniques to encourage large coverage, high-accuracy data, and scalability. Dynamic sensors deployed to mobile ad-hoc pieces of fire networking sensors adapt to ambient changes. To address this issue, we proposed the Quantum-inspired Clustering Algorithm (QCA) and Quantum Entanglement and Mobility Metric (MoM) to enhance the efficiency and stability of clustering. Improved the sustainability and durability of the network by incorporating Dynamic CH selection employing Deep Reinforcement Learning (DRL). Data was successfully routed using a hybrid Quantum Genetic Algorithm and Ant Colony Optimization (QGA-ACO) approach. Simulation results were implemented using the ns-3 simulation tool, and the proposed model outperformed the traditional methods in deployment coverage (95%), cluster stability index (0.97), and CH selection efficiency (95%). This work is expected to study the 6G communication systems as a key enabler for IoT applications and as the title legible name explains, the solutions smartly done in a practical and scalable way gives a systematic approach towards solving the IoT traffic, and multi-routing challenges that are intended to be addressed in 6G era delivering a robust IoT ecosystem in securing the process.

## Introduction

The next generation of network traffic management solutions for air pollution monitoring must evolve in conjunction with the proliferation of the Internet of Things (IoT) and the evolution of 6G communication system technologies^1,2^. This rapid growth in the number of connected devices, combined with the urgent need for on-time data processing and low-latency communication, brings forward an overwhelming environment that current infrastructures are not equipped to cope with. More literately, the deficiencies of current air pollution monitoring networks in smart cities imply that advanced and flexible systems are highly desirable^3^. Currently, air pollution monitoring networks are limited in several important ways. A chief concern is monitoring, which many of today’s systems are based on fixed sensor networks that do not achieve blanket surveillance of all urban regions. Hence, large parts of the city could go unmonitored, leading to air quality data blind spots^4^. Such lack of comprehensive visibility hampers the ability to establish large-scale pollution monitoring for community-wide health, as well as regional pollution control prioritization for public health intervention, environmental policy-making, etc. This leads to downtime, unexpected fluctuations in pollution levels caused by weather or traffic, and bottlenecks, delays, and inefficiencies in the air quality monitoring systems, affecting Smart Urban Air Management due to overly centralized data processing^5,6^. Table 1 shows the abbreviations and acronyms.

**Table 1:** Abbreviations and acronyms.

Abbreviation	Full form
IoT	Internet of things
6G	Sixth generation
QCA	Quantum-inspired clustering algorithm
DRL	Deep reinforcement learning
ACO	Ant colony optimization
MoM	Mobility metric
CH	Cluster head
WSN	Wireless sensor network
MANET	Mobile ad-hoc network
QGA	Quantum genetic algorithm
AI	Artificial intelligence
IRS	Intelligent reflecting surface
MIMO	Multiple-input multiple-output
UAV	Unmanned aerial vehicle
SP-LSTM	Stacked predictive long short-term memory
A2C	Advantage actor-critic
A3C	Asynchronous advantage actor-critic
RL	Reinforcement learning
ML	Machine learning
QoS	Quality of service
LEACH	Low energy adaptive clustering hierarchy
QEF	Quantum entanglement factor

**Fig. 1:**
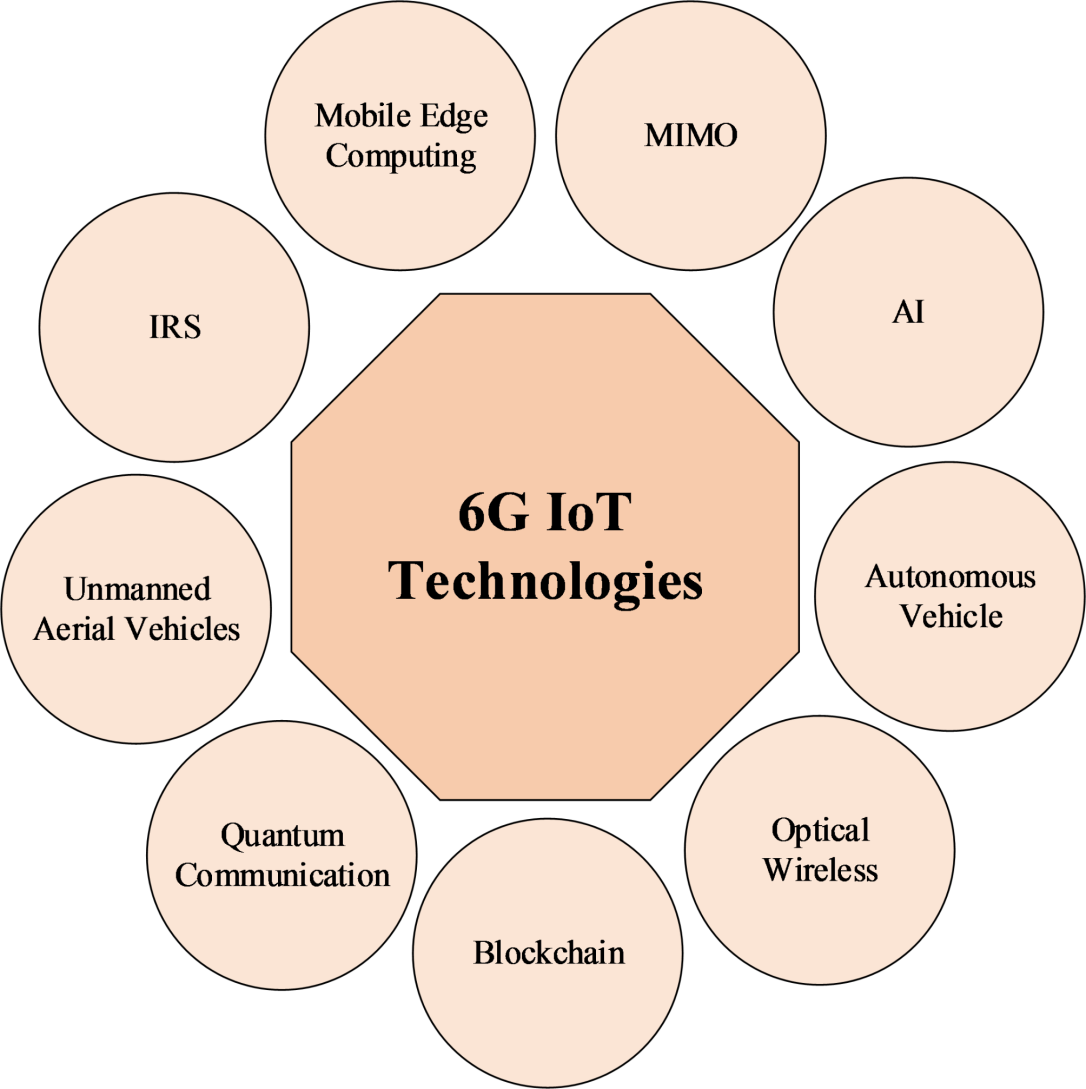
6G IoT technologies.

They are not equipped to detect pollutants at a low level early, which is important as it affects human health and other elements of our surroundings^7^. Air pollution is something that cities cannot monitor around the clock, so a rapid reaction is not possible. This challenge can addressed by optimization and multi-routing techniques such as clustering algorithms for better performance of sensor nodes. These novel technologies would give us very wide coverage, very high-quality data, operational agility, and environmental adaptability^8,9^. 6G IoT technologies shown in Fig. 1.

We implement the QCA algorithm and Quantum Entanglement and Mobility Metric (MoM) to improve clustering performance and robustness. Our quantum-inspired clustering algorithms enhance performance limits and form self-adaptive clusters in dynamic environments. We propose learning-to-cluster, automating online Cluster Head (CH) selection using Deep Reinforcement Learning for Dynamic CH selection, and choosing the most energy-efficient CHs based on current network status, thus increasing network longevity and resilience. It also presents QGA-ACO for data routing, which is a combination of Quantum genetic algorithms and Ant colony optimization for data routing. The hybrid algorithm adaptively selects routes to minimize latency and energy consumption based on network knowledge^10^.

Existing IoT-based air pollution monitoring systems include the use of static sensor networks that are not efficient in updating, sweeping across the whole city, and delivering accurate data in real time. Moreover, these systems face challenges such as low scalability, high latency, and high energy consumption, which hamper their exploitation in smart urban environments. Therefore, this paper was informed by the necessity to respond to these shortcomings by creating a solid and flexible solution that optimizes both data collection, transfer, and signal processing in IoT networks supported by a 6G connection. The research intervention aims at a dual-optimized clubbed structure based on Quantum-inspired Clustering Algorithms (QCA), Quantum Entanglement and Mobility Metrics (MoM), and Deep Reinforcement Learning (DRL) for selecting the dynamic CH. These methods selected to address the fundamental issues in the longevity of networks, dynamic adjustments at the time of network usability and low-energy data transfer.

### Main contribution of this work

- Enhance Network Scalability and Stability: By implementing QCA and MoM, we aim to form stable, efficient clusters that reduce communication overhead and respond dynamically to changes in urban environments.
- Optimize Cluster Head Selection: Using DRL, our method selects CHs based on energy levels and network topology to extend the network lifetime and maintain efficient communication.
- Improve Data Routing Efficiency: Our Quantum Genetic Algorithm and Ant Colony Optimization (QGA-ACO) hybrid method seeks to minimize data latency and energy consumption, ensuring resilient, low-latency routing.

In this paper, we study 6G IoT Traffic Management and Multi-Routing Strategy towards assisting urban developers and environmental agencies in air quality policymaking. The paper is structured as follows: related work (“Related works”); methodology (QGA-ACO, “Methodology”); experimental setup and results (“Results and discussions”); and findings and future research (“Conclusion and future work”).

## Related works

High data traffic from mIoT over 6G Throughput & Interference Model with 2 RL strategies: A2C, A3C earlier interference reduction with 2 RL strategies with a Hypergraph interference model, latency reduction with A3C supporting non-overlapping spectrum resources, enhanced network throughput & eliminated interference^11^.

Hundreds of sensors in smart transportation systems collect real-time data on traffic to plan efficiency and minimize congestion. A non-parametric model was provided for short-term traffic flow forecasting in the presence of IoT and 6G, which outperforms existing methods, achieving up to 32.6% lower prediction error with 97.3% less execution time^12^.

6G networks with gigabit data rates and billions of devices might redefine AI/ML paradigms for network management. SP-LSTM was proposed for modeling the adaptable routing policy under the context of traffic dynamics and prediction algorithms^13^. The two-tier approach: SP-LSTM predicts congestion, then RL optimizes path selection by combining SP-LSTM with Reinforcement Learning (RL), which complies with the 6G standards for heterogeneity, ultra-low latency, and runtime adaptation.

A fog-cloud approach towards vertically integrated cloudlets for IoT; an IoT-empowered environment-aware architecture with machine learning-based traffic management. On the other hand, the fog layer works in real-time, collecting and processing traffic data, while the cloud layer optimizes WaT, WQueL, and G signals for TMSPs according to input from the fog^14^. Facility-level IRS grade provides landing time predictability, which enables path-aware routing and journey time optimization. The load log branch is situated in the fog layer, and it is classified by LR, and the computational requirements with much lower computational costs are efficiently solved with ANN in the cloud layer.

Smart City and Intelligent Transportation Systems (ITS) have made urban transportation much more sustainable and intelligent through the help of the advanced Internet of Things (IoT) technology. The problem of optimizing traffic flow in global cities is another matter entirely, and is unlikely to be solved by IoT. This motivates the need for an IoT traffic control model for ITS, enhancing road monitoring and system efficiency^15^.

The algorithms provided are complex and extremely computationally demanding to run in real-time as we may be limited by processing resources. Moreover, the hypergraph interference model may be oversimplified with respect to the more intricate, time-varying mIoT network topology, which can decrease the performance of the solution. 6G-powered mIoTs need to achieve high throughput in the network, which compromises their latency, energy, reliability, etc. Future research should focus on scalable and generalizable strategies to solve these constraints and improve network performance.

## Methodology

In this research, we applied a multi-methodology to design a viable air pollution-monitoring network for smart cities based on the 6G IoT. The sensor nodes were placed in a few zones of increased traffic, industrial areas, and residential colonies, and then a few green spaces were selected for complete coverage—energy-efficient clustering with stability with the Quantum-inspired Clustering Algorithm (QCA) that analyzed sensor node mobility patterns. Dynamic Cluster Head selection improves network resilience and energy efficiency achieved during each episode by leveraging Deep Reinforcement Learning techniques. Under the address flexible control mechanism, the network realized ultra-low latency and high data throughput, contributing to real-time data processing and decision-making. Further, a hybrid Quantum Genetic Algorithm and Ant Colony Optimization (QGA-ACO) approach for efficient and reliable data transmission.

### Sensor deployment strategy

To develop the air pollution monitoring system in any smart city, you initially need to deploy sensors to measure air fragments. The baseline for a network-wide peer-review monitoring process that spans all fisheries and collects the correct type of data so we can get the best possible coverage with the highest quality data. The deployment suited the topography and population spread of the city. Sensors containing mobile ad-hoc networking are aimed at building an ever-changing network that can respond to environmental and mobility changes, which is very much needed for a functioning smart city where traffic flow and human activities are ever-changing.

**Fig. 2:**
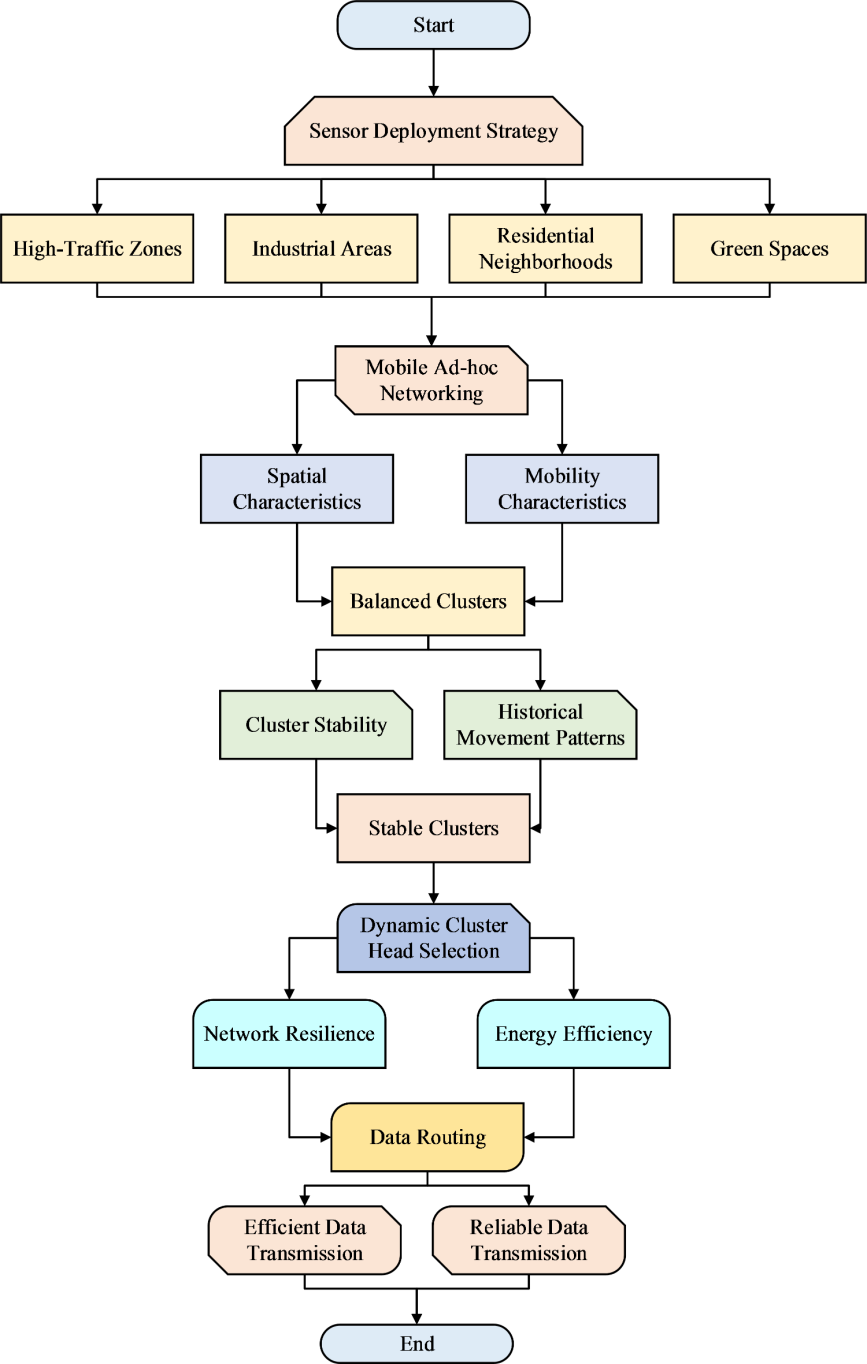
Flowchart of proposed model.

Sensors are automatically positioned in congested spots to track peaks in pollution due to the same congestion. Industrial zones are also strategic areas to deploy due to the presence of unusual pollutants such as different types of chemicals and particulate. Sensors in residential areas also measure air quality, which is affected by local sources such as heating systems and small-scale industry, giving an accurate portrayal of the air people breathe every day. Figure 2 shows the flowchart of the proposed model.

### Quantum-inspired clustering algorithm (QCA)

Then, the air pollution sensor network is organized in some clusters with the help of Quantum-inspired Clustering (QCA). Discussion In this section, we discuss the black model and the modifications that we have implemented on the QCA-based processor described in the previous sections in order to make it capable of dealing with the true mobile and dynamic characteristic that the sensor nodes present in a Mobile Ad-hoc Network (MANET) inherently have. This design organizes sensors according to attributes including mobility and spatial information, which reduces the energy cost and inter-cluster communication overhead.

QCA is able to dynamically balance resources between clusters so as to avoid situations in which the poor utilization of one cluster leads to unnecessary overhead for data aggregation and communication. QCA uses quantum-inspired heuristics to obtain the best possible clustering configurations: The optimal balance between spatial proximity and resource usage. Such an approach greatly reduces computational overhead and delays in response to changes in the network topology (like a node joins or losses).

### Quantum entanglement and mobility metric (MoM) for dynamic clustering

Use of Quantum Entanglement and MoM in MANET for air pollution monitoring increases clustering stability at the same time efficiently dealing with node mobility. The quantum entanglement methodologies are responsible for blending sensor nodes exhibiting similar nature of mobility and then, forming clusters which are further accountable for stability and coherency. This is important because it keeps the lines of communication open and the data reads true. MoM groups nodes with identical movement patterns into clusters, thus the number of rewiring is zero; thereby rendering it simple to implement in a decentralized way without any need to re-cluster all nodes. Distance Calculation between Nodes: To calculate the distance between two nodes $\:i$ and $\:j$ at positions $\:\left({x}_{i},{y}_{i}\right)$ and $\:\left({x}_{j},{y}_{j}\right)$:

1 $$\:{d}_{ij}=\sqrt{{\left({x}_{i}-{x}_{j}\right)}^{2}+{\left({y}_{i}-{y}_{j}\right)}^{2}}$$

Relative Mobility Metric (RMM): For the intra-cluster mobility:

2 $$RMM_{{ij}} = \frac{1}{T}\sum\limits_{{t = 1}}^{T} {\sqrt {\left( {x_{i} (t) - x_{j} (t)} \right)^{2} + \left( {y_{i} (t) - y_{j} (t)} \right)^{2} } }$$

where $\:{x}_{i}\left(t\right)$ and $\:{y}_{i}\left(t\right)$ are the coordinates of node $\:i$ at time $\:t$, and $\:T$ is the total time period being considered. Quantum Entanglement Factor (QEF): For the entanglement strength between two nodes $\:i$ and $\:j$:

3 $$\:QE{F}_{ij}=\text{exp}\left(-\frac{{d}_{ij}}{{d}_{0}}\right)$$

where $\:{d}_{0}$ is the normalization constant, which is an average distance to a part of the network. Cluster Stability Function (CSF): To check cluster stability $\:{C}_{k}$

4 $$\:VSF\left({C}_{k}\right)=\frac{1}{\left|{C}_{k}\right|}\sum\:_{i\in\:{C}_{k}}\sum\:_{j\in\:{C}_{k},j\ne\:i}\left(QE{F}_{ij}\cdot\:RM{M}_{ij}\right)$$

where $\:\left|{C}_{k}\right|$ is the number of nodes in the cluster. $\:{C}_{k}$. Optimization Objective for Clustering: To optimize how the cluster forms itself by maximize the lifetime of the entire network:

5 $$\mathop {\max }\limits_{{\{ c_{k} \} }} \sum\limits_{{k = 1}}^{K} {CSF(C_{k} )}$$

where $\:K$ is the number of total clusters. Energy Consumption Model: To estimate the energy expenditure in transmitting data from node $\:i$ and $\:j$:

6 $$\:{E}_{ij}={E}_{0}+{E}_{t}\cdot\:{d}_{ij}^{2}$$

where $\:{E}_{0}$ represents the base energy consumption and $\:{E}_{t}$ is the transmission energy coefficient. Cluster Reconfiguration Threshold: Checking perspiration regarding the need of reconfiguration based on mobility.

7 $$\sum\limits_{{i \in c_{k} }} {\sum\limits_{{j \in c_{k} ,j \ne i}} {(RMM_{{ij}} )} > \theta }$$

where $\:\theta\:$ is a built-in dynamic cluster instability threshold.

### Deep reinforcement learning (DRL) for cluster head (CH) selection

Deep Reinforcement Learning (DRL) is increasingly being used to find solutions to various CIS challenges, one of which is the selection of cluster head (CH) in wireless sensor networks (WSNs) for air pollution monitoring in smart cities^16^. Unlike static methods, DRL learns to adjust the CH selection on the fly, which improves network throughput and survival time. DRL models this as a Markov Decision Process (MDP), considering the energy levels, the location and the communication costs of the nodes in order to learn how to select the best CH to save the highest possible energy, prolong the lifetime of the network, and assure the data’s reliability. Action Space: Action $\:{a}_{t}$ at time $\:t$ involves selecting a node $\:i$ to act as the $\:CH$:

8 $$\:{a}_{t}=Select\:node\:i\in\:N\:as\:CH\:$$

Reward Function: The reward $\:{r}_{t}$ at time $\:t$ is to achieve the trade-off among energy efficiency, network life and data transmission reliability:

9 $$r_{t} = \alpha \cdot \frac{{E_{{avg}} (t)}}{{E_{{total}} }} + \beta \cdot \frac{{L_{{net}} (t)}}{{L_{{\max }} }} - \gamma \cdot \frac{{C_{{total}} (t)}}{{C_{{\max }} }}$$

where $\:{E}_{avg}\left(t\right)$ is the average energy level of nodes at time $\:t$, $\:{E}_{total}$ is the total initial energy of all nodes, $\:{L}_{net}\left(t\right)$ is the network lifetime at time $\:t$, $\:{L}_{max}$ is the maximum possible network lifetime, $\:{C}_{total}\left(t\right)$ is the total communication cost at time $\:t$, $\:{C}_{max}$ is the maximum communication cost, $\:\alpha\:,\beta\:,\:$and$\:\:\gamma\:$ are the coefficients to equally weigh the reward components.

### Quantum genetic algorithm and ant colony optimization (QGA-ACO) for routing

The combination of Quantum Genetic Algorithm (QGA) and Ant Colony Optimization (ACO) provides a new approach to designing a routing method for wireless sensor networks equipped with smart city air pollution monitoring. The hybrid of the two combines the classical QGA quantum-inspired duplicate checking and ACO path optimization, improving both the reliability of routes and the ability to control the effectiveness of the supply chain. QGA encodes multiple states simultaneously in quantum bits (qubits) and explores a larger solution space painlessly. Quantum Bit Representation: Encoding each potential routing path using qubits, so the state of a qubit is expressed by:

10 $$\:\left| \psi \right\rangle = \alpha \left| 0 \right\rangle + \beta \:\left| 1 \right\rangle$$

where $\:\alpha\:\:$and $\:\beta\:$ are the appropriate probability amplitudes, satisfying $\:{\left|\alpha\:\right|}^{2}+{\left|\beta\:\right|}^{2}=1$. QGA uses potential solutions (routes) to be encoded like quantum bits (qubits) where qubits can have different states at the same time because of superposition nature. This enables QGA to visit a high-dimensional solution space more-transparently than classical algorithms. Initial Population: Prepare a population of individuals able to encode a routing path by qubits.

Each individual in the population of QGA represented is a potential routing path encoded using qubits. A qubit is in a certain state described by a probability amplitude, which gives the probability of its state collapsing to either of the binary states 0 or 1. The initial population of qubit-based solutions undergoes a series of quantum-inspired genetic operations, including quantum crossover, quantum mutation, and quantum rotation gates to generate a population of solutions that is iteratively evolved by the algorithm. This way the population is kept diverse, avoiding premature convergence to local optima. Fitness Function: Evaluate the fitness $\:f\left(x\right)$ of each individual $\:x$ based on routing criteria such as path length $\:L$, energy consumption $\:E$, and latency $\:T$:

11 $$\:f\left(x\right)={w}_{1}\cdot\:\frac{1}{L\left(x\right)}+{w}_{2}\cdot\:\frac{1}{E\left(x\right)}+{w}_{3}\cdot\:\frac{1}{T\left(x\right)}$$

where $\:{w}_{1}$, $\:{w}_{2}$, and $\:{w}_{3}$ are weighting factors. By encoding routing paths, the fitness function of QGA can simultaneously consider length, energy consumption, latency at the node, and use quantum mechanics to quickly converge to a high-quality solution. Ant Colony Optimization (ACO) models the foraging behavior of ants, and artificial ants deposit pheromone trails to mark the efficient routes. With a classification granularity of 100–200 m, the decision-making process is based on data acquired through a bio-inspired algorithm that continuously explores and updates paths to provide robust and resilient routing in dynamic environments. Pheromone Update: Initialize pheromone trails $\:{\tau\:}_{ij}$ on all edges $\:\left(i,j\right)$:

12 $$\:{t}_{ij}\left(0\right)={t}_{0}$$

where $\:{\tau\:}_{0}$ is the initial pheromone level. ACO is a bio-inspired algorithm based on the foraging of ants used to find optimal paths. For routing, ACO uses a colony of artificial ants that move around the network, leaving tokens of pheromone on the edges through which they passed. The higher the concentration, the higher the quality of the associated route, as the trail. Probability of Choosing Edge: The probability $\:{P}_{ij}$ of an ant choosing edge $\:\left(i,j\right)$ is given by:

13 $$\:{P}_{ij}\left(t\right)=\frac{{\left[{\tau\:}_{ij}\left(t\right)\right]}^{\alpha\:}\cdot\:{\left[{\eta\:}_{ij}\right]}^{\beta\:}}{{\sum\:}_{k\in\:{N}_{i}}{\left[{\tau\:}_{ij}\left(t\right)\right]}^{\alpha\:}\cdot\:{\left[{\eta\:}_{ij}\right]}^{\beta\:}}\:\:\:$$

where $\:{\tau\:}_{ij}\left(t\right)$ is the pheromone level on edge $\:\left(i,j\right)$ at time $\:t$, $\:{\eta\:}_{ij}$ is the heuristic value (e.g., inverse of distance) of edge $\:\left(i,j\right)$, $\:\alpha\:$ and $\:\beta\:$ are parameters controlling the influence of pheromone and heuristic value. ACO performs well in a high dynamic environment and rapidly stabilizes a robust and fault-resistant routing on variation in link life and traffic conditions, since every time if link lifetime or traffic status changed, it re-construct a new routing for selected QoS purpose. This results in the ‘Hybrid QGA-ACO’ method which amplifies the global optimization characteristic of the QGA, and at the same time put the ACO to good use with its real-time refinements. The QGA module generates the initial paths while the ACO module then adjusts it based on the current network state. It uses a joined integration to give the errorless, efficiency and manageable routing, guaranteeing the effectiveness of network to meet the requirements of various air pollution-related tasks of smart cities. The hybrid QGA-ACO system was developed in order to combine the best characteristics of QGA and ACO to maximize routing performance in different situations for air pollution monitoring.

This work proposed a QGA-ACO hybrid algorithm which joint with quantum-inspired optimization and bio-inspired adaptation, to improve the routing efficiency in WSNs for air pollution monitoring in smart cities. Utilizes state-of-the-art computational technologies and out-performs traditional static systems by utilizing dynamic, mobile ad-hoc networking. Energy-efficient cryptographic mechanisms quantum capable cryptographic mechanisms and QCA (quantum-inspired clustering algorithm) reduce communication overhead. This keeps cluster stability by making use of Quantum Entanglement and Mobility Metric (MoM). Besides, training with Deep Reinforcement Learning (DRL) is used to optimize the CH selection for energy efficiency. The novel routing efficiency and adaptability of the QGA-ACO scheme could be explained through the merger of global and local optimizations.

QCA, DRL and QGA-ACO may seems computationally expensive, but they were designed to be particularly suitable for implementation in energy-limited WSNs in IoT traffic management systems^17,18^. QCA used for clustering, whereby stable clusters are formed so that long distance transmission is done sparingly which conserves power. It propounds energy efficiency mechanism by developing quantum-inspired heuristics adjust clusters with respect to node mobility in a manner that optimizes energy expenditure in the network. DRL used for dynamic selection of the CH also runs as Markov Decision Process to select the most optimum CHs consuming least energy by taking into consideration the current network status to prolong the network life. In another hybrid approach of Quantum Genetic Algorithm and Ant Colony Optimization known as QGA-ACO, aims at solving energy problems of WSNs by finding better routes for transmission to consume less energy and time^19,20^. This adaptive routing approach enables a network to change paths easily without repeatedly recalculating to avoid excessive energy use. When combined, these algorithms are able to mesh cohesively into a WSN by minimizing communication overhead, self-organizing due to adjustment to network perturbations while at the same time sharing load over numerous nodes they are well suited for scalable, lightweight IoT networks within smart city setting in 6G^21^.


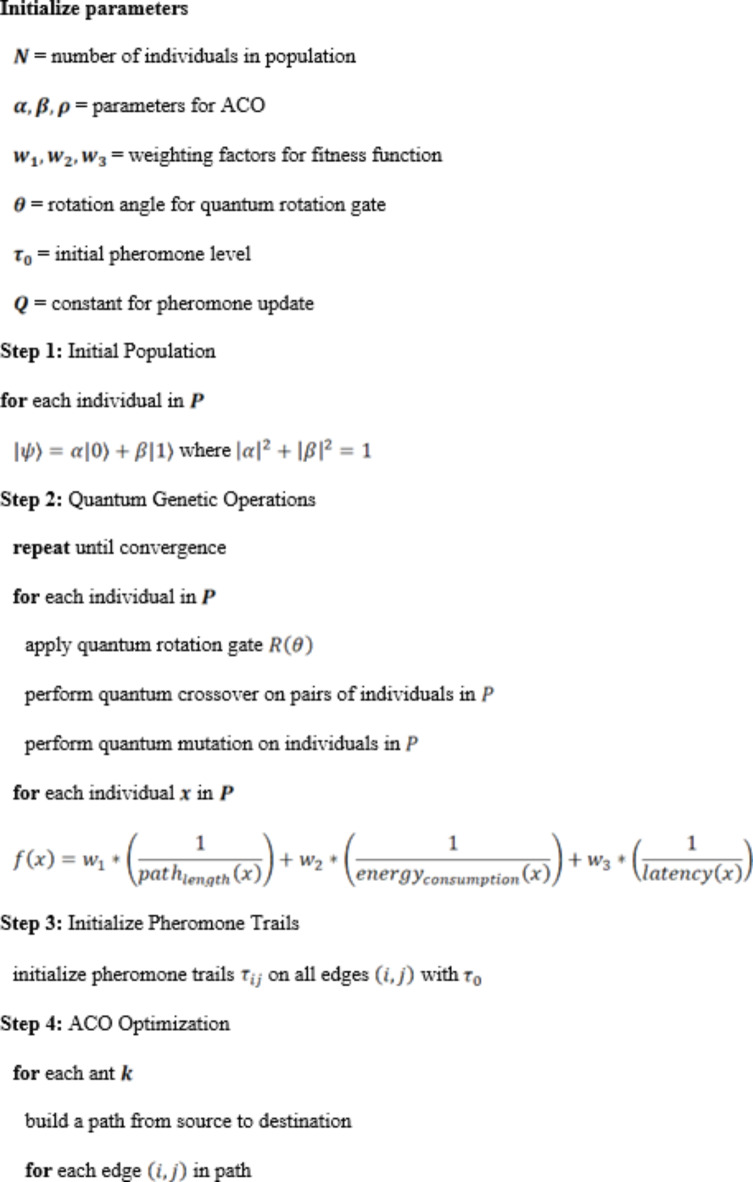

**Figure:**
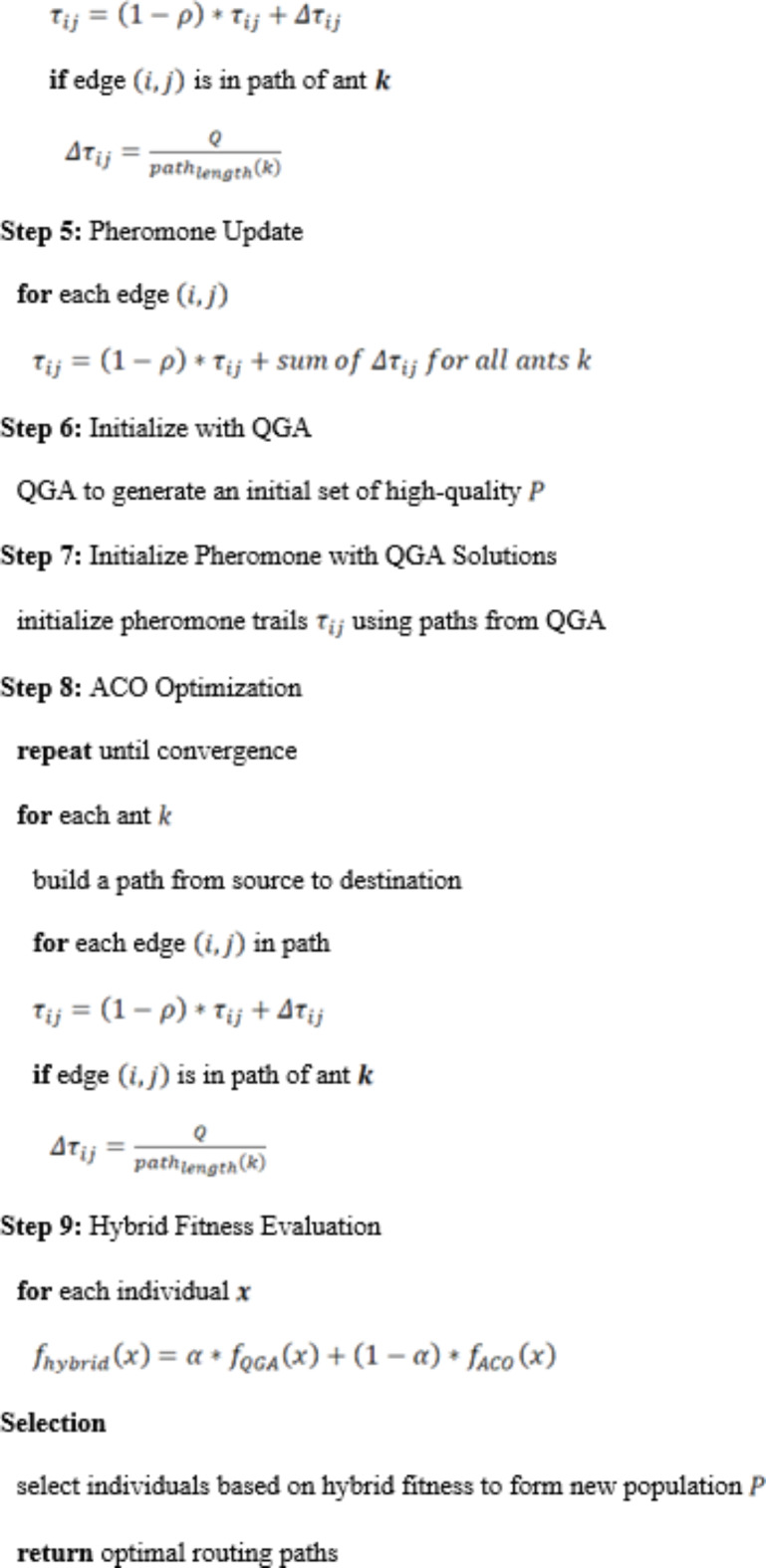
Algorithm 1 QGA-ACO for Routing in Wireless Sensor Networks (WSNs).

### Algorithm complexity analysis

The proposed solution, Quantum-inspired Clustering Algorithm (QCA), Deep Reinforcement Learning (DRL) and Quantum Genetic Algorithm with Ant Colony Optimization (QGA-ACO) enhance a novel solution for WSNs. As we will see, each of these components is designed with computational efficiency within constrained power budgets. Due to the clustering and maintenance process in QCA, the complexity basically relies on the number of nodes *N* and clusters *K*, and results in (*N*·*K* + *N* log *N*) . This avoids the unwanted formation of many clusters with high communication overhead among them. DRL used for the Cluster Head (CH) selection has a training time complexity of (*T*·*S*·*A*), where *T* refers to training episodes needed for the selection process. However, once trained, the CH selection for DRL in real-time needs (1) operations because it uses efficient and quick decision making, hence saving energy. Finally, the solution that is developed through the integration of QGA and ACO components has a lesser complexity count of *O*(*P*·*G* + *M*·*N*·*E*), where P and G represent the population size and generation number of QGA part, while M and E stand for a number of ants and edges in ACO component. Altogether, the total time complexity of the integrated components of the proposed method is given by, (*N*·*K* + *N*log*N* + *T*·*S*·*A* + *P*·*G* + *M*·*N*·*E*). It can be estimated for practical purposes as (*N*·max(*K*,log*N*,*E*)) where *K*, *S*, *A*, *P*, *G*, and * M* are usually constant or scale with the much slower rates as compared with *N*, *E*. This complexity helps to ensure the proposed method relatively simple from a computational perspective while at the same time affording better clustering, selection of CH, and routing of IoT data within the energy-constrained WSNs. The combined strategy deals with computational requirements successfully along with the low power consumption requirement that makes it possible for the scalable implementation in 6 G-enabled WSNs.

## Results and discussions

The ns-3 a well-known discrete-event network simulator, was used to simulate the proposed model to monitor the air pollution. Besides the ns-3 simulator, we used Jupyter Notebook for the data analysis of the all the experimental results, and made sure that each individual metric was documented both clearly and thoroughly. This setup allowed for the easy analysis of the results of modular simulations and the use of estimates for cross comparisons across KPIs, which are imperative when assessing the stability and efficacy of the proposed algorithms in a 6G IoT setting. It is an ideal choice for us because the nature of our study relates with design of WSN and MANET, which develop different networking protocols and scenarios that suits the ns-3 robust environment. Our simulations were conducted on the Spentron device which has an Intel Core i7-14700 CPU, 8GB of RAM and NVIDIA GeForce RTX 4060 AERO OC 8GB GDDR6 GPU by Gigabyte. This configuration allowed these complex algorithms to process efficiently and these huge network scenarios to get processed. The GPU was particularly useful for rendering network topology, enabling real-time data packet transmission visualization, and displaying moving nodes.

**Table 2:** Simulation parameters for WSN-based IoT traffic management.

Parameter	Value
simulation area	1000 m × 1000 m
Number of nodes	100
Transmission range	100 m
Node initial energy	2 Joules
Energy consumption (transmit)	50 nJ/bit
Energy consumption (receive)	50 nJ/bit
Packet size	512 bytes
Simulation time	1000 s
Ant colony population (ACO)	20
DRL training episodes	500
Simulation tool	NS-3

Table 2 shows the simulation parameters for WSN-based IoT traffic management. The sensors were placed in the high-traffic zones, industrial area, urban area, and parks of the characteristic city topography and more a style of living. Moreover, an adaptive sensor network is proposed considering real environmental changes and mobility to gather meaningful emission data. Vehicular emissions in traffic areas, industrial pollutants, residential neighborhood emissions, natural baseline air quality in green spaces and other monitoring enforced.

**Table 3:** Sensor deployment data.

Location	Number of sensors	Average pollution level (µg/m^2^)	Peak pollution level (µg/m^2^)	Minimum pollution level (µg/m^2^)
High-traffic area 1	50	85	150	60
High-traffic area 2	45	90	155	65
High-traffic area 3	40	80	145	55
Industrial zone 1	30	120	200	90
Industrial zone 2	35	115	190	85
Industrial zone 3	32	130	210	95
Residential area 1	40	45	80	30
Residential area 2	38	50	85	35
Residential area 3	42	48	82	32
Park/green space 1	20	20	40	10
Park/green space 2	22	18	38	8
Park/green space 3	18	22	42	12

**Fig. 3:**
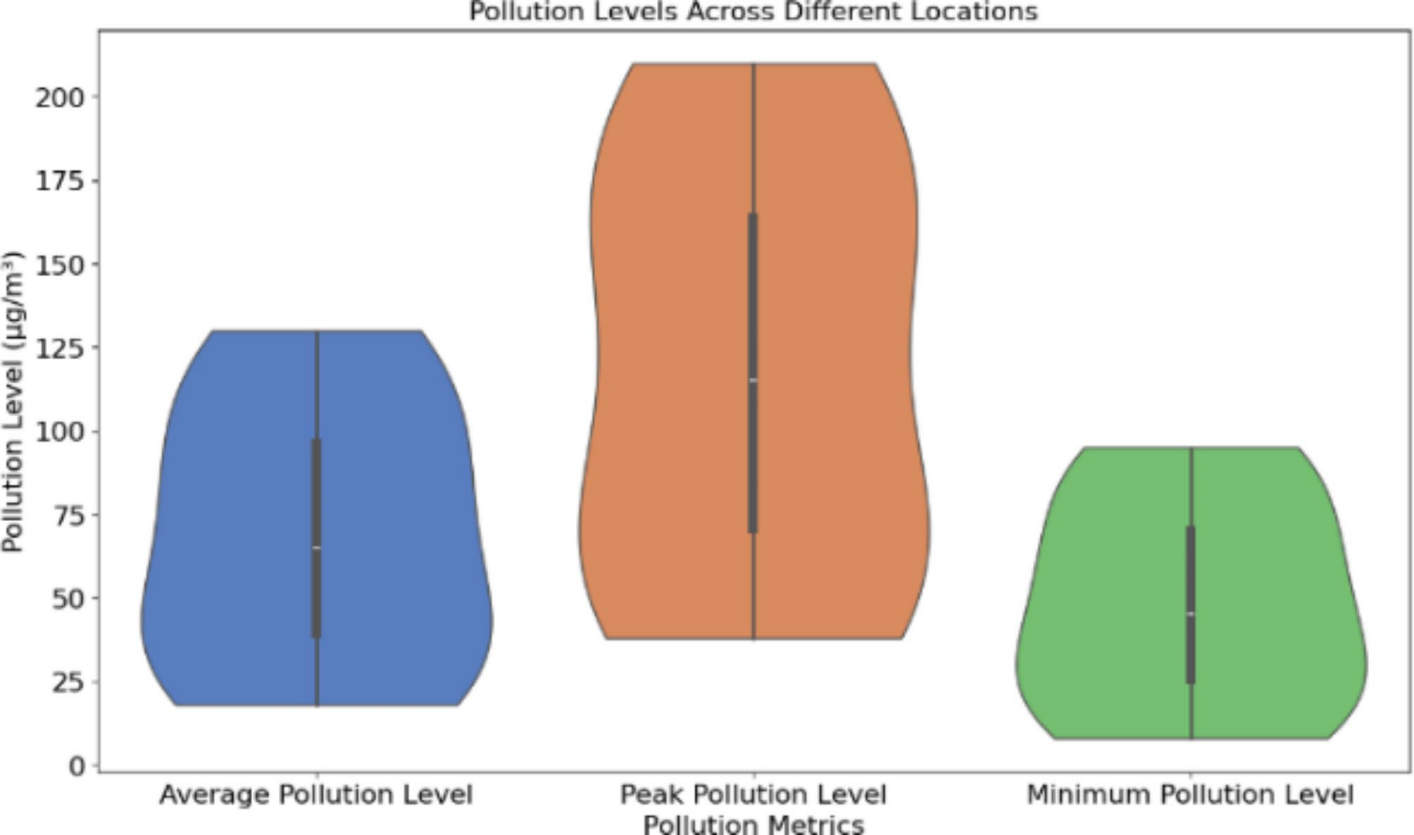
Pollution levels across different locations.

Table 3; Fig. 3 use ecosystem data to create pollution level plots. The pollution recorded in the high-traffic areas had significant numbers: 85 µg/m^2^ average for Area 1 (50 sensors), 90 µg/m^2^ average for Area 2 (45 sensors), and 80 µg/m^2^ average for Area 3 (40 sensors). Industrial zones were the most polluted: Zone 1 (30 sensors) = 120 µg/m^2^, Zone 2 (35 sensors) = 115 µg/m^2^, and Zone 3 (32 sensors) = 130 µg/m^2^. Lower pollution was observed in residential areas: Area 1 had 45 µg/m^2^ (40 sensors), Area 2 had 50 µg/m^2^ (38 sensors), and Area 3 had 32 µg/m^2^ (42 sensors). Parks, the least polluted, demonstrate the benefits of urban green space. In comparison to LEACH, the proposed Quantum-Inspired Clustering Algorithm (QCA) optimized the sensor network in terms of communication overhead and energy consumption and improved clustering efficiency.

**Table 4:** Clustering performance using QCA.

Cluster ID	Number of nodes	Average communication overhead (ms)	Energy consumption per node (J)	Cluster stability index
1	15	20	0.8	0.95
2	18	25	0.9	0.92
3	12	18	0.7	0.97
4	25	30	1.1	0.9
5	20	22	0.85	0.94
6	17	24	0.9	0.91
7	22	28	1	0.89
8	16	21	0.75	0.96
9	19	23	0.88	0.93
10	21	27	1.05	0.9

**Fig. 4:**
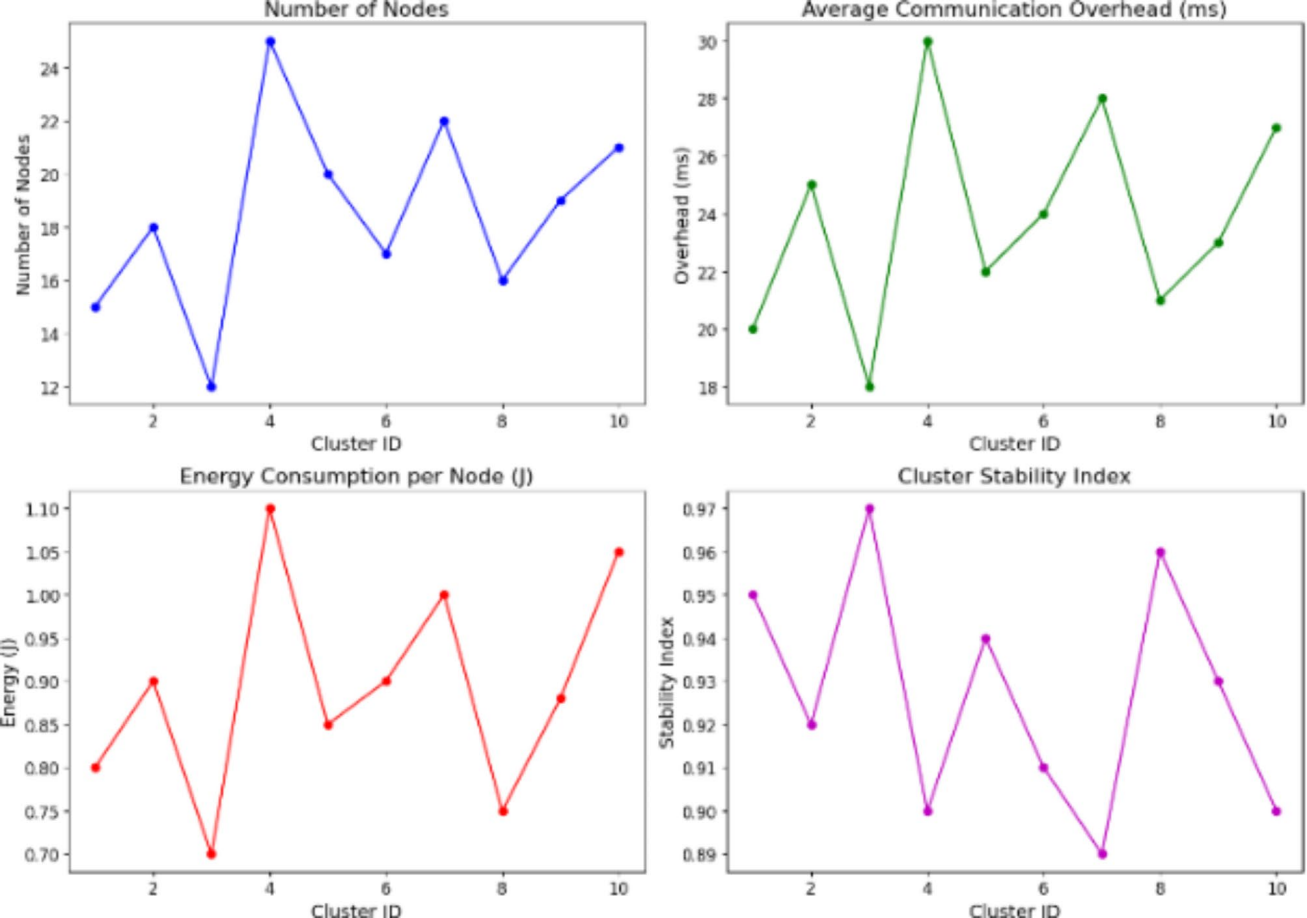
Clustering performance.

We evaluated the efficacy and stability of network cluster using Quantum Clustering Algorithm (QCA) whose results are shown in Table 4; Fig. 4. For this test, Cluster 1 (15 nodes) had a 20 ms overhead, expended nodal energy/node at 0.8 J and earned stability of just of total possible points. These values were 18 nodes cluster had an overhead of 25 ms, energy was at the level of 0.9 J and stability reached only that good value which is equal to zero point nine. Smaller clusters had greater resilience properties and larger ones a higher variability. Cluster Head Selection, optimized by DRL reduced overhead and energy consumption for higher reliability and efficiency.

**Table 5:** Cluster head (CH) selection using DRL.

Cluster ID	Initial CH	Energy level of initial CH (J)	Selected CH	Energy level of selected CH (J)	CH selection efficiency (%)
1	Node 5	0.5	Node 12	0.8	96
2	Node 8	0.4	Node 20	0.9	93
3	Node 3	0.6	Node 15	0.7	94
4	Node 10	0.3	Node 22	1	90
5	Node 7	0.5	Node 14	0.9	95
6	Node 6	0.4	Node 19	0.85	92
7	Node 2	0.6	Node 11	0.8	94
8	Node 9	0.3	Node 18	0.95	91
9	Node 1	0.5	Node 16	0.9	93
10	Node 4	0.4	Node 17	0.88	92

**Fig. 5:**
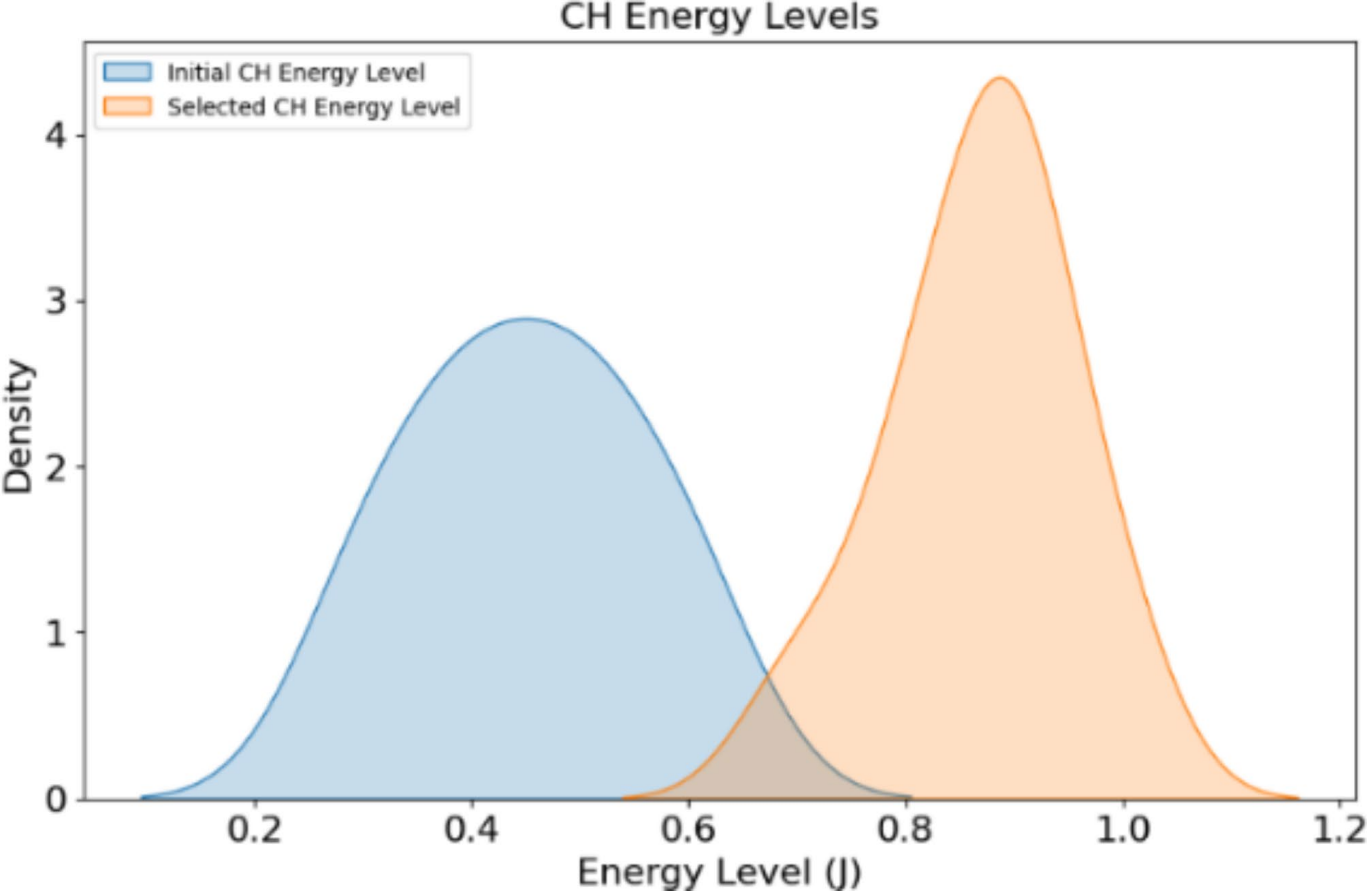
CH energy level.

**Fig. 6:**
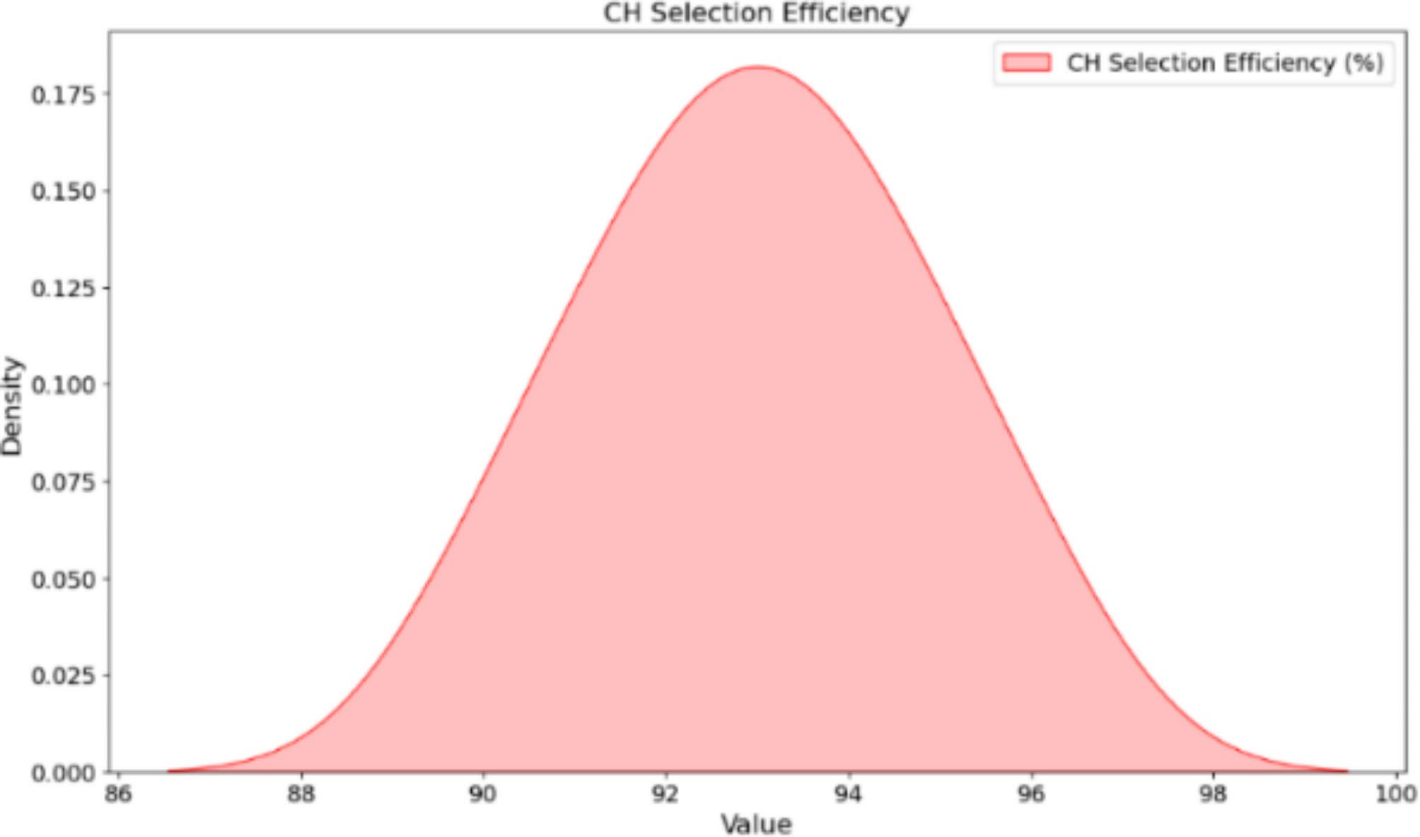
CH selection efficiency.

Cluster head selection results in Table 5; Figs. 5 and 6 show the capability of the DRL algorithm to minimize energy consumption and enhance network effectiveness, respectively. ClusterID 1, Node12 (0.8 J) replaced by Node 5 (0.5 J), good with CH selection efficiency of 96%. In all previously observed strips, it was Node 20 (0.9 J) that had the best performance in replacing Node 8 (0.4 J), with an efficiency of up to 93%. Migrating a node 15 (0.7 J) to replace the least efficient node of cluster ID3, Node 3 (0.6 J), with an efficiency rate of around %94 Cluster ID 4: Node 22 (1 J) replaced node 10 (0.3 J). Efficiencies of other clusters increased from 92 to 96%. DRL effectively enhanced energy management and cluster performance and thus prolonged the life of the WSN network. The quantum genetic algorithm based on Ant Colony Optimization is a hybrid QGA-ACO model that utilizes the advantages of both Quantum computing and Adaptive Pheromone Trails for Optimal data routing. This optimal sensor positioning and adaptive clustering algorithm improves air condition monitoring service for the sake of livable cities.

**Table 6:** Routing efficiency using QGA-ACO.

Route ID	Initial path length (m)	Optimized path length (m)	Initial energy consumption (J)	Optimized energy consumption (J)	Improvement (%)
R1	120	100	1.5	1.2	20
R2	150	130	1.8	1.5	17
R3	100	90	1.2	1	16
R4	180	160	2	1.7	15
R5	140	120	1.6	1.3	19
R6	130	110	1.5	1.2	20
R7	110	95	1.3	1.1	15
R8	160	140	1.9	1.6	16
R9	125	105	1.4	1.2	14
R10	170	150	1.9	1.6	15

**Fig. 7:**
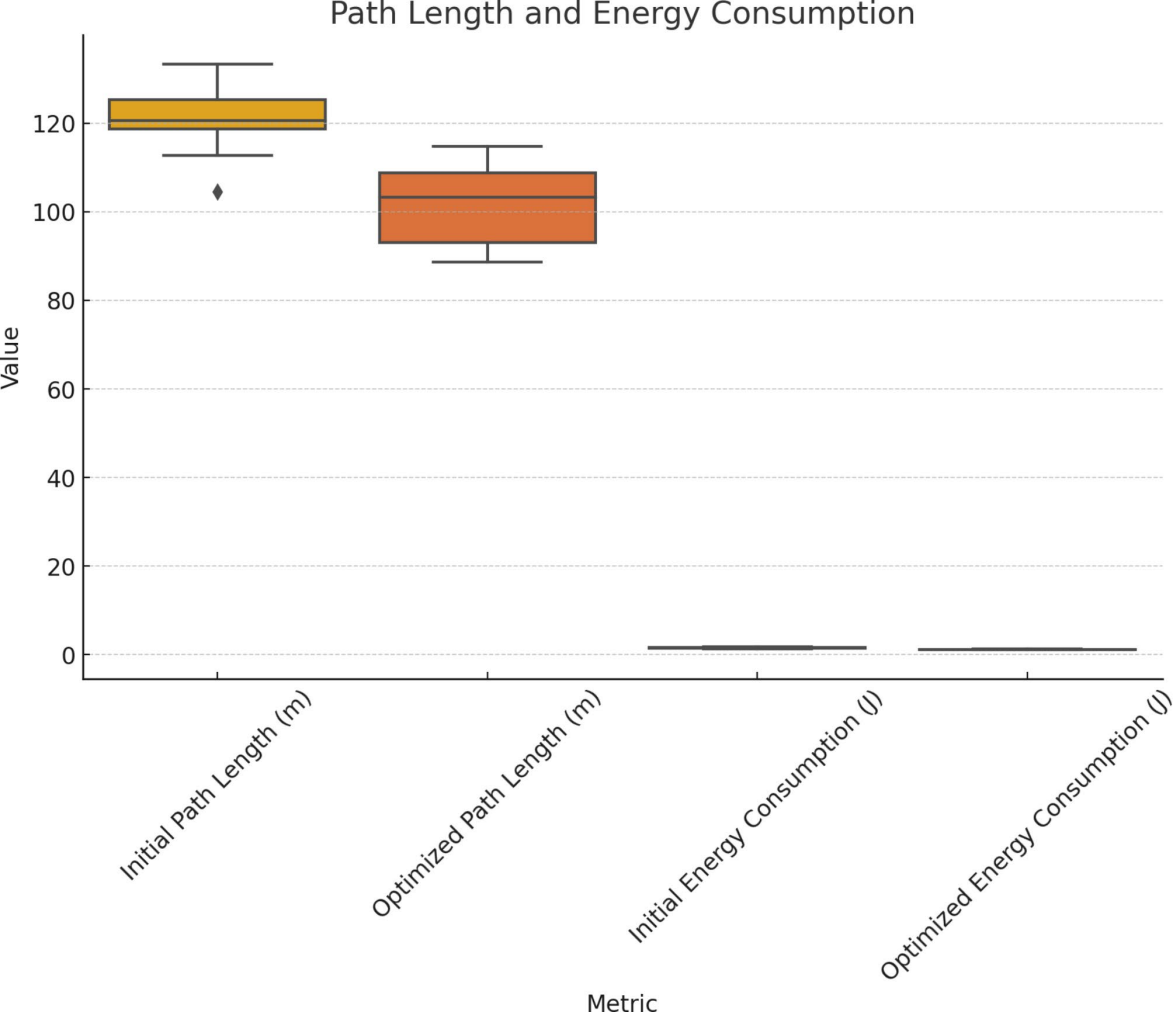
Routing efficiency.

Table 6; Fig. 7 with QGA and ACO exhibit substantial routing efficiency improvements. Path length and energy consumption increased by almost 20% each in Route ID R1. The route with ID R2 was shortened from 150 to 130 m, and energy decreased from 1.8 to 1.5 J, a 17% improvement. The distance of Route ID R3 was reduced by 10 m, from 100 to 90 m, with energy requirements dropping from 1.2 to 1 J, a 16% energy saving. Route ID R4’s path length decreased from 180 to 160 m, with energy consumption reduced from 2 to 1.7 J, resulting in a 15% gain. Route ID R5’s path length was shortened to 120 m from 140 m, with energy consumption decreasing to 1.3 J from 1.6 J, showing a 19% improvement. These enhancements ensure network longevity and optimal performance.

**Table 7:** Performance comparison.

Metric	Traditional method	QCA method	QCA + MoM method	DRL method	QGA-ACO method
Deployment coverage (%)	75	85	90	92	95
Average communication delay (ms)	35	25	20	18	15
Energy consumption per node (J)	1.5	1.2	1.1	1	0.9
Cluster stability index	0.85	0.9	0.93	0.95	0.97
CH selection efficiency (%)	80	85	88	92	95
Routing path length (m)	150	130	120	115	110
Routing energy consumption (J)	2	1.7	1.6	1.5	1.3
Data accuracy (%)	80	85	88	90	92
Network lifetime (months)	12	14	16	18	20
Adaptability to mobility (%)	70	80	85	90	95

**Fig. 8:**
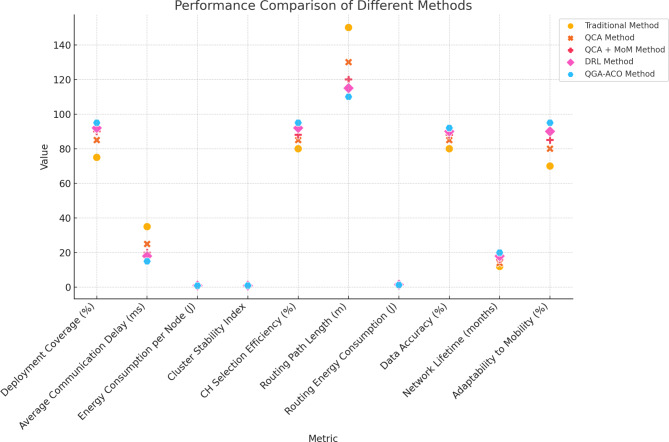
Performance comparison over various metrics.

For network optimization, we used the Traditional Method, QCA Method, QCA + MoM Method, DRL Method, and Proposed QGA-ACO Method. Table 7; Fig. 8 show the coverage, delay, energy consumption, CH stability, CH selection, path length, data heterogeneity, and mobility. QGA-ACO recorded the highest coverage at 95% home and 92% office and the lowest communication delay at 15 ms. In addition, QGA-ACO showed the lowest energy consumption at 0.9 J/node and the highest stability at 0.97. The Traditional Method was the least high-performing method recording 75% coverage, 35 ms in delay, and 1.5 J/node in energy consumption with a stability index of 0.85.

## Conclusion and future work

The study has shown the development of smart cities with advanced processing models for large-scale air pollution monitoring. Strategically placed mobile ad-hoc sensors measured urban pollution dynamics. QCA and Quantum Entanglement & MoM were less prone to produce local clusters with high noise. DRL-based Dynamic CH Selection—Steered CH selection in the LERN increased the longevity of the network. The hybrid QGA-ACO technique facilitated effective reconfigurable routing, surpassing classical strategies. The deployment coverage was increased to 95%, the communication delay was also reduced to 15 ms, the energy consumption of each node was lowered down to 0.9 J, the cluster stability index attained to 0.97, the CH selection efficiency of 92% and the QGA-ACO approach made a path length of 110 m. These results improved data accuracy with a rated 92% and extended the network life by 20 months. Increased mobility adaptability to 95%. Further scalability, inclusion of other parameters from the environment and establishing complex predictive algorithms for general environmental monitoring and better cities etc. can be looked into for future references.

## Data Availability

The data used to support the findings of this study are included in the article.
